# Knowledge, attitudes, and practices regarding China’s DRG payment policy among healthcare professionals in tertiary and secondary hospitals in Yunnan Province: a cross-sectional study

**DOI:** 10.3389/fpubh.2025.1685544

**Published:** 2025-11-03

**Authors:** Jian Yang, Dan Qin, Fan Li

**Affiliations:** 1 School of Pharmaceutical Sciences & Yunnan Provincial Key Laboratory of Pharmacology for Natural Products, Kunming Medical University, Kunming, Yunnan, China; 2 Yunnan Provincial Center for Drug Policy Research, Kunming, Yunnan, China; 3 College of Modern Biomedical Industry, Kunming Medical University, Kunming, Yunnan, China; 4 Technology Transfer Center, Kunming Medical University, Kunming, Yunnan, China

**Keywords:** CHS-DRG payment policy, value-based healthcare, knowledge, attitudes, and practices (KAP), structural equation modeling, hospital management, health policy

## Abstract

**Background:**

The China Healthcare Security Diagnosis Related Groups (CHS-DRG) payment policy is a cornerstone of China’s transition to value-based healthcare. Its successful implementation hinges on the knowledge, attitudes, and practices (KAP) of healthcare professionals, particularly in the large hospitals that are the main arenas for this reform. This study aimed to examine the KAP of healthcare professionals in tertiary and secondary hospitals in Yunnan Province and to analyze the interrelationships between these core constructs.

**Methods:**

A cross-sectional survey was conducted from March to May 2023 among 357 healthcare professionals from 30 tertiary and secondary hospitals in Yunnan Province. A self-administered questionnaire was used to collect data on demographics and KAP regarding the CHS-DRG policy. Structural equation modeling (SEM) was employed to test the hypothesized relationships between knowledge, attitudes, and practices, while analysis of variance (ANOVA) was used to explore differences across demographic groups.

**Results:**

The study found that healthcare professionals possessed moderate knowledge (mean score: 4.01/6) and practice levels, but held generally positive attitudes (mean score: 13.39/18) toward the CHS-DRG policy. The SEM analysis revealed that attitudes (*β* = 0.462, *p* < 0.001) had a stronger direct effect on practices than knowledge (*β* = 0.360, *p* < 0.001), and that attitudes partially mediated the relationship between knowledge and practice. Furthermore, demographic factors such as age, work experience, and education level were found to have significant, nuanced impacts on specific dimensions of KAP.

**Conclusion:**

In the main battleground of China’s DRG reform—tertiary and secondary hospitals—healthcare professionals’ attitudes are a more potent driver of their implementation practices than their formal knowledge. While enhancing policy knowledge is necessary, fostering a positive organizational climate that addresses professional concerns is critical. Implementation strategies should be tailored to the specific needs of different demographic subgroups to effectively translate policy into practice.

## 1 Introduction

China is undertaking a profound transformation of its healthcare system, shifting from a volume-based, fee-for-service model to a value-based system focused on quality and efficiency while reducing the patient’s financial burden (1). A central pillar of this reform is the nationwide implementation of the China Healthcare Security Diagnosis Related Groups (CHS-DRG) payment policy, initiated in 2019 (2). This system, which reimburses hospitals based on patient diagnoses and treatment complexity rather than the volume of services, is China’s adaptation of a globally recognized tool for promoting value-based healthcare, following similar principles to DRG systems long established in the United States and Europe (3). The policy’s goal is to curb the excessive healthcare service utilization incentivized by the traditional fee-for-service model and to align financial rewards with quality and safety outcomes (4).

The success of such a large-scale policy reform is not guaranteed; it heavily depends on the acceptance and adoption by frontline healthcare professionals (5). As seen internationally, the implementation of DRG-based systems often encounters challenges, and the CHS-DRG is no exception (6). Common barriers include a limited understanding of complex coding and documentation requirements, resistance to altering established clinical habits, and significant concerns over the policy’s financial implications for both institutions and individuals (7).

To understand these dynamics, the Knowledge, Attitudes, and Practices (KAP) framework provides a foundational model. It posits that knowledge is the basis for forming attitudes, which in turn drives behavior (8). However, to address criticisms of the KAP model’s potential oversimplification and to enhance theoretical depth, this study integrates insights from organizational behavior theories (9). In the complex, high-pressure environment of a hospital, an individual’s behavior is shaped by more than just personal knowledge and attitudes (10). Factors such as perceived social pressure from colleagues and leaders (subjective norms) and confidence in one’s ability to perform the new tasks (perceived behavioral control) are also critical. This integrated theoretical lens allows for a more nuanced exploration of the factors mediating the path from policy knowledge to practical implementation (11).

This study focuses on Yunnan Province, a region in Southwest China characterized by significant ethnic diversity and uneven economic development. Its healthcare system presents a unique microcosm for observing the rollout of national policies in non-metropolitan, resource-constrained settings (12). The current phase of CHS-DRG implementation is concentrated in tertiary and secondary hospitals, which have the necessary infrastructure and handle the majority of complex cases. These institutions are the primary battleground where the policy’s intended effects and unintended consequences first materialize (13). Therefore, examining the KAP of professionals within these key hospitals offers a critical, targeted assessment of the policy’s real-world impact. By focusing on this core group, the study aims to generate deep insights into the challenges and opportunities at the forefront of this major reform (14).

This study aims to assess the current levels of knowledge, attitudes, and practices regarding the CHS-DRG policy among healthcare professionals in tertiary and secondary hospitals in Yunnan Province. Furthermore, the research will utilize structural equation modeling (SEM) to test a hypothesized KAP model and examine the direct and indirect relationships between these three constructs. Ultimately, the study seeks to identify the key factors influencing the implementation of the CHS-DRG policy to provide evidence-based recommendations for policymakers and hospital administrators, thereby facilitating the policy’s successful implementation across China.

## 2 Methods

### 2.1 Research design and participants

A cross-sectional study was conducted between March and May 2023 in Yunnan Province, China. Eligible participants were healthcare professionals with at least 6 months of work experience in hospitals actively implementing the CHS-DRG payment policy. Individuals on vacation or sick leave during the study period were excluded.

### 2.2 Sampling methodology

A multi-stage sampling methodology was employed to ensure a representative sample. First, six cities within Yunnan Province (Kunming, Qujing, Yuxi, Dali, Honghe, and Wenshan) were selected based on their diverse economic development levels and population distribution. Second, within these cities, a stratified sampling approach was used to select 30 hospitals. Inclusion criteria for hospitals stipulated that they must be tertiary or secondary public institutions that had officially implemented the CHS-DRG payment system for at least 6 months. Hospitals were selected from each city’s official lists to ensure representation across both institutional tiers.

The study’s design deliberately focused on tertiary and secondary hospitals, a decision based on the current implementation landscape of the CHS-DRG policy in China. These larger hospitals are the primary sites for the policy’s rollout, as they possess the necessary case-mix complexity and health information systems. They are the frontline organizations where the policy’s impact is most direct and significant. By concentrating on this population, this study provides a targeted and in-depth analysis of the policy’s core challenges and successes. Finally, within each selected hospital, participants were recruited from various departments to ensure a diverse sample of physicians, pharmacists, nurses, and medical insurance staff. A total of 357 valid questionnaires were collected.

### 2.3 Sample size determination

The sample size was determined using the single population proportion formula: n = Z_α/2_^2^ * P * (1-P) / d^2^. For this calculation, we used parameters based on a prior study in other Chinese provinces, which reported a 54.3% prevalence (P) of CHS-DRG knowledge, along with a 95% confidence interval (Z_α/₂_ = 1.96) and a 5% margin of error (d = 0.05) (15). The initial calculation for a large population yielded a sample size of 381. As our study targeted a specific, finite population of healthcare professionals within the selected hospitals, an adjustment was made. After adding a 10% contingency for non-responses, the final minimum target sample size was established at 245. The 357 valid responses obtained in this study comfortably exceeded this minimum, ensuring sufficient statistical power for the subsequent structural equation model analyses.

### 2.4 Questionnaire and variables

A self-administered questionnaire was utilized to collect data on the knowledge, attitudes, and practices of healthcare professionals regarding the CHS-DRG payment policy. To ensure content validity and relevance, the questionnaire was developed based on a comprehensive literature review and refined through consultation with experts in health policy and hospital management. The instrument consisted of four distinct sections: (1) demographic information of the participants; (2) a knowledge section with six questions to assess understanding of the policy, where one point was awarded for each correct answer for a total score ranging from 0 to 6; (3) an attitudes section comprising six questions to gauge perceptions of the policy’s impact, rated on a three-point scale with total scores ranging from 6 to 18; and (4) a practices section with seven questions to measure the application of the policy in daily work, with scores assigned based on option levels for a total score range of 7 to 28.

### 2.5 Research hypotheses

Based on an integrated theoretical framework combining the traditional Knowledge, Attitudes, and Practices (KAP) model with insights from organizational behavior theories, this study proposed several hypotheses to test the relationships among variables. The core KAP pathway suggests that knowledge is the foundation for attitude formation, which in turn drives practices. Accordingly, it was hypothesized that: (H1) Knowledge of the CHS-DRG payment policy positively impacts attitudes toward it; (H2) Positive attitudes toward the policy positively impact implementation practices; and (H3) Knowledge of the policy has a direct positive impact on implementation practices.

### 2.6 Data analysis

Data were analyzed using SPSS 27.0 and AMOS 26.0. First, descriptive statistics were used to summarize participant characteristics. The psychometric properties of the questionnaire’s Knowledge, Attitudes, and Practices sections were assessed using Cronbach’s *α*, Confirmatory Factor Analysis (CFA), Average Variance Extracted (AVE), and Composite Reliability (CR). Second, to explore the influence of demographic characteristics on KAP, independent samples t-tests and one-way analysis of variance (ANOVA) were employed. Finally, structural equation modeling (SEM) was used to test the hypothesized relationships between the core constructs of knowledge, attitudes, and practices. The fit of the structural model was evaluated using several indices: chi-square/degrees of freedom (χ^2^/df < 5), Comparative Fit Index (CFI > 0.90), Tucker–Lewis Index (TLI > 0.90), and Root Mean Square Error of Approximation (RMSEA < 0.08). A *p*-value < 0.05 was considered statistically significant for all tests.

## 3 Results

### 3.1 Participant characteristics

**Figure 1:**
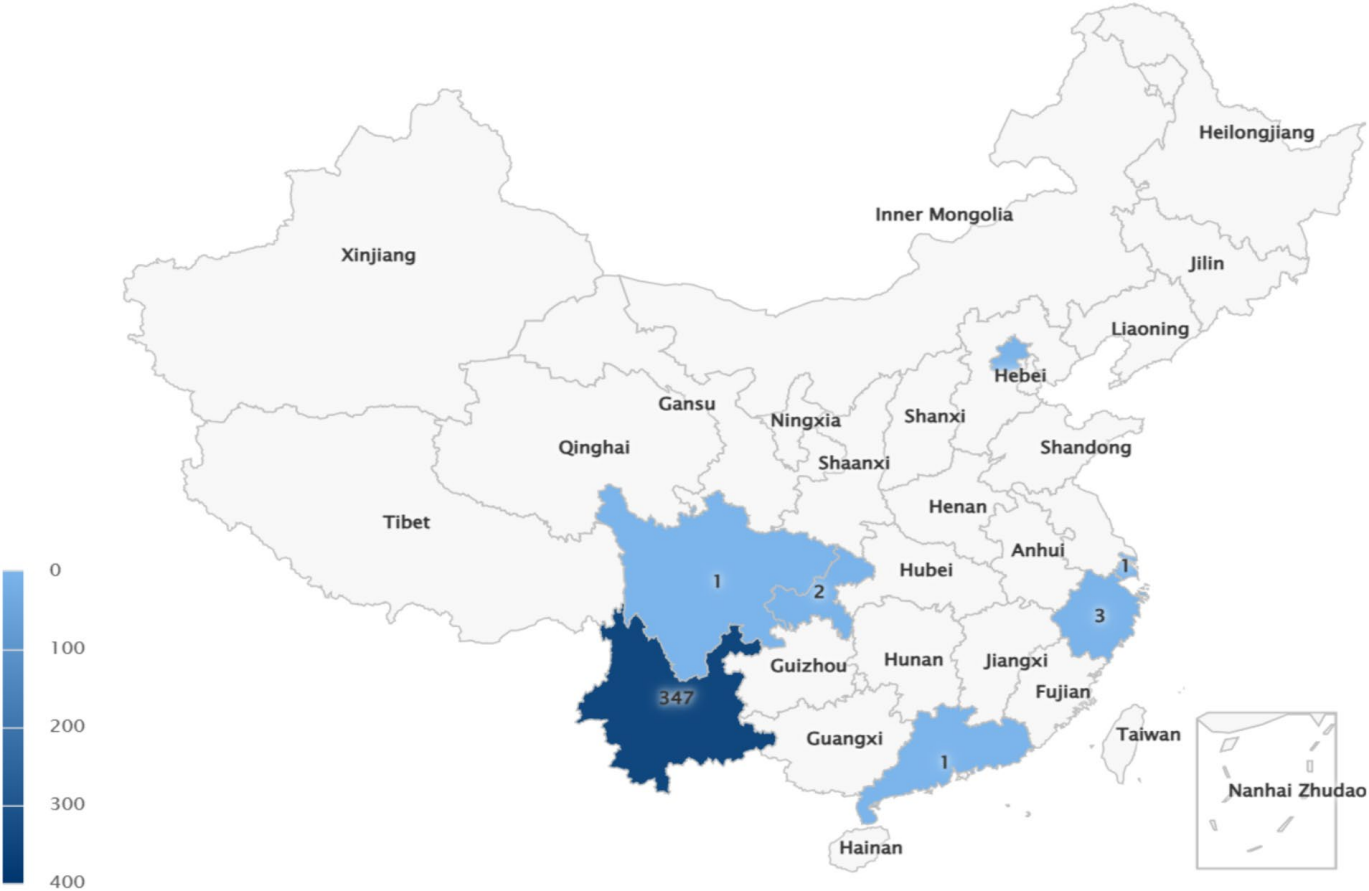
Survey object distribution map.

A total of 357 healthcare professionals participated in this study. The sample was predominantly female (68.6%), with nearly half of the participants (49.3%) aged between 26 and 35 years. The primary professional roles represented were clinical pharmacists (39.5%), physicians (26.1%), and nurses (21.3%). All participants held a college degree or higher, with a majority (74.8%) possessing an undergraduate degree. In terms of professional titles, 31.1% were at the intermediate level. As intended by the study’s focus, participants were primarily from tertiary (59.4%) and secondary (34.7%) hospitals. The self-developed questionnaire demonstrated good internal consistency (Cronbach’s *α* = 0.838) and construct validity (KMO = 0.894). Detailed demographic characteristics of the participants are presented in Table 1 (see Figure 1).

**Table 1:** Demographic characteristics of the participants.

Variables	Variables	*n*	%
Gender	Male	112	31.4
	Female	245	68.6
Age (years)	<25	31	8.7
	26–35	176	49.3
	36–45	93	26.0
	>45	57	16.0
Professional title	Senior Title / Chief (e.g., Chief Physician)	16	4.5
	Associate Senior Title / Associate Chief (e.g., Associate Chief Physician)	46	12.9
	Intermediate Title (e.g., Attending Physician)	111	31.1
	Junior Title / Assistant (e.g., Resident Physician)	104	29.1
	Trainee / Intern	39	10.9
	Other	41	11.5
Position	Doctor	93	26.1
	Pharmacist	141	39.5
	Nurse	76	21.3
	Technician	20	5.6
	Medical insurance department staff	14	3.9
	Other	13	3.6
Education level	Master’s degree and above	37	10.4
	Undergraduate	267	74.8
	Specialist	53	14.8
Hospital nature	Third level comprehensive hospitals (including traditional Chinese medicine hospitals)	212	59.4
	Secondary comprehensive hospitals (including traditional Chinese medicine hospitals)	124	34.7
	Traditional Chinese Medicine Specialized Hospital	12	3.43
	Community Health Service Center (including service stations)	2	0.6
	Township health centers (including clinics)	6	1.7
	Other	1	0.3
Years of work	0–1 years	9	2.5
	2–5 years	82	23.0
	6–10 years	110	30.8
	11–15 years	62	17.4
	16 years and above	94	26.3

### 3.2 Descriptive scores of knowledge, attitudes, and practices

Overall, participants demonstrated moderate knowledge (mean score: 4.01/6) and practice levels (mean score: 18.59/28), alongside generally positive attitudes toward the CHS-DRG policy (mean score: 13.39/18), as shown in Table 2. To further explore these findings, a detailed comparative analysis was conducted to examine how KAP scores varied across different demographic characteristics. The results of this analysis are presented in the subsequent section.

**Table 2:** Mean scores and standard deviations of knowledge, attitudes, and practices.

Variables	Mean	SD
Knowledge	4.01	2.80
Attitudes	13.39	3.38
Practices	18.59	6.41

### 3.3 Analysis of KAP scores by demographic characteristics

A bivariate analysis was performed to identify significant differences in KAP scores among various subgroups (Table 3). The analysis revealed that knowledge scores were significantly associated with several professional factors. Years of work had a highly significant influence (*F* = 7.486, *p* < 0.001), with professionals having 16 or more years of experience demonstrating the highest level of knowledge. Similarly, age was a significant factor (*F* = 5.710, *p* < 0.001), where older participants scored higher. The respondent’s position also had a significant impact (*F* = 5.978, *p* < 0.001), with pharmacists, in particular, showing superior knowledge scores compared to other roles. Finally, hospital nature was found to significantly affect knowledge levels (*F* = 3.247, *p* = 0.007). In contrast, attitude scores showed limited variation across demographic groups. The only factor with a statistically significant influence was education level (*F* = 6.422, *p* = 0.002). Interestingly, professionals with specialist-level (college) degrees reported less positive attitudes (mean score: 12.41) than their counterparts with undergraduate (13.37) or postgraduate degrees (14.13). Practice scores were influenced by a wide range of professional characteristics. Significant differences were observed based on education level (*F* = 8.441, *p* < 0.001), position (*F* = 4.439, *p* < 0.001), professional title (*F* = 3.432, *p* = 0.005), and hospital nature (*F* = 2.978, *p* = 0.012). For instance, professionals with senior titles reported higher practice scores, while those with postgraduate degrees reported lower practice scores compared to those with specialist and undergraduate qualifications. Gender, age, and years of work did not show a significant association with practice scores.

**Table 3:** Analysis of differences in knowledge, attitude, and practice (KAP) scores by demographic characteristics.

Variables	Variables	K			A			*p*		
		M ± SD	F/t	*p*	M ± SD	F/t	*p*	M ± SD	F/t	*p*
Gender	Male	4.13 ± 2.32	−0.627	0.531	13.43 ± 2.64	−0.231	0.818	21.40 ± 5.82	−0.657	0.512
	Female	3.96 ± 2.40			13.36 ± 2.11			21.01 ± 4.97		
Age (years)	<25	3.71 ± 2.67	5.710	< 0.001	13.90 ± 2.41	1.801	0.147	22.71 ± 5.68	1.343	0.260
	26–35	3.59 ± 2.49			13.27 ± 2.17			20.85 ± 5.35		
	36–45	4.32 ± 2.19			13.14 ± 2.52			20.85 ± 4.94		
	>45	4.95 ± 1.77			13.84 ± 2.12			21.60 ± 5.14		
Professional title	Senior Title / Chief (e.g., Chief Physician)	4.10 ± 2.48	0.997	0.419	13.51 ± 2.01	1.404	0.222	22.88 ± 4.79	3.432	0.005
	Associate Senior Title / Associate Chief (e.g., Associate Chief Physician)	4.31 ± 2.61			14.03 ± 2.47			22.72 ± 5.18		
	Intermediate Title (e.g., Attending Physician)	3.71 ± 2.59			13.43 ± 2.14			21.50 ± 5.56		
	Junior Title / Assistant (e.g., Resident Physician)	3.89 ± 2.20			13.21 ± 2.49			19.80 ± 5.09		
	Trainee / Intern	4.41 ± 2.08			12.87 ± 2.30			21.02 ± 4.65		
	Other	4.63 ± 1.96			13.88 ± 1.67			19.94 ± 5.18		
Position	Doctor	4.80 ± 1.97	5.978	<0.001	13.71 ± 2.24	0.571	0.722	22.76 ± 4.92	4.439	<0.001
	Pharmacist	5.71 ± 0.73			13.64 ± 1.65			22.92 ± 3.60		
	Nurse	2.75 ± 2.69			13.55 ± 2.21			22.25 ± 5.00		
	Technician	3.62 ± 2.66			13.31 ± 2.43			19.69 ± 6.18		
	Medical insurance department staff	4.16 ± 2.20			13.31 ± 2.06			21.57 ± 5.30		
	Other	3.52 ± 2.51			13.16 ± 2.71			19.76 ± 5.15		
Education level	Master’s degree and above	4.45 ± 2.27	2.247	0.107	14.13 ± 2.55	6.422	0.022	22.87 ± 5.48	8.441	<0.001
	Undergraduate	4.01 ± 2.42			13.37 ± 2.19			21.17 ± 5.12		
	Specialist	3.38 ± 2.05			12.41 ± 2.27			18.35 ± 4.75		
Hospital nature	Third level comprehensive hospitals (including traditional Chinese medicine hospitals)	4.03 ± 2.31	3.247	0.007	13.17 ± 2.22	1.748	0.123	20.51 ± 4.94	2.978	0.012
	Secondary comprehensive hospitals (including traditional Chinese medicine hospitals)	4.26 ± 2.28			13.74 ± 2.80			22.30 ± 5.14		
	Traditional Chinese Medicine Specialized Hospital	2.92 ± 3.06			14.08 ± 2.31			21.92 ± 6.67		
	Community Health Service Center (including service stations)	3.00 ± 4.24			14.50 ± 3.54			20.50 ± 16.26		
	Township health centers (including clinics)	1.17 ± 2.40			12.17 ± 3.49			19.50 ± 7.20		
Years of work	0–1 years	2.67 ± 2.96	7.486	<0.001	13.33 ± 1.32	0.867	0.484	22.11 ± 5.46	1.638	0.164
	2–5 years	3.43 ± 2.68			13.37 ± 2.21			21.59 ± 5.54		
	6–10 years	3.70 ± 2.36			13.29 ± 2.21			20.61 ± 5.37		
	11–15 years	3.94 ± 2.37			13.06 ± 2.48			20.11 ± 5.00		
	16 years and above	5.05 ± 1.64			13.72 ± 2.38			21.93 ± 4.90		

### 3.4 Measurement model analysis

Prior to testing the structural model, the validity and reliability of the measurement model were established using Confirmatory Factor Analysis (CFA). This analysis focused on the three core latent constructs: Knowledge, Attitudes, and Practices. The results, detailed in Tables 4, 5, indicated that the measurement model had an acceptable fit with the data. The analysis of construct reliability and convergent validity showed strong results for two of the three constructs. The ‘Knowledge’ (CR = 0.922, AVE = 0.667) and ‘Practices’ (CR = 0.898, AVE = 0.564) constructs both exceeded the recommended thresholds of 0.7 for Composite Reliability (CR) and 0.5 for Average Variance Extracted (AVE), demonstrating strong internal consistency and convergent validity. For the ‘Attitudes’ construct, the Composite Reliability was robust (CR = 0.794), indicating good internal consistency among its items. However, its Average Variance Extracted (AVE = 0.453) was slightly below the 0.5 threshold. Following established guidelines, a construct with an AVE below 0.5 is considered acceptable if its CR remains above 0.6. Given the strong CR value, the ‘Attitudes’ construct was retained for the subsequent structural model analysis. Overall, the measurement model was deemed sufficiently robust to proceed with hypothesis testing.

**Table 4:** Latent variables and observed variables.

Latent variables	Items	Observed variables
Knowledge	K1	Ever understood of CHS-DRG payment policy
	K2	Principal characteristics of CHS-DRG payment policy
	K3	Knowledge regarding the scope of applicability of CHS-DRG payment policy
	K4	Utilization methods of CHS-DRG payment
	K5	CHS-DRG payment policy impacts medical practices
	K6	Training related to the CHS-DRG payment policy
Attitudes	A1	Attitudes toward the CHS-DRG payment policy
	A2	Enhance healthcare quality by CHS-DRG payment policy
	A3	Increase the workload by CHS-DRG payment policy
	A4	Reduce healthcare costs by CHS-DRG payment policy
	A5	Improve patient satisfaction by CHS-DRG payment policy
	A6	Enhance the efficiency of medical practices by CHS-DRG payment policy
Practices	P1	Usage of the CHS-DRG payment policy in daily work
	P2	Consider the CHS-DRG payment policy in decision-making process
	P3	Provide feedback on the implementation of the CHS-DRG payment policy
	P4	Communicate with patients and their families regarding issues related to the CHS-DRG payment policy
	P5	Participate in discussions or training related to the CHS-DRG payment policy
	P6	Strictly adhere to the CHS-DRG payment policy
	P8	Confidence and ability to effectively implement the CHS-DRG payment policy

**Table 5:** Measurement model: construct reliability and convergent validity.

Latent construct	Observed variables (items)	Standardized factor loadings	Composite Reliability (CR)	Average Variance Extracted (AVE)
Knowledge	K1	0.773	**0.922**	**0.667**
	K2	0.874		
	K3	0.881		
	K4	0.863		
	K5	0.771		
	K6	0.721		
Attitudes	A1	0.727	**0.794**	**0.453**
	A2	0.807		
	A3	−0.063		
	A4	0.476		
	A5	0.826		
	A6	0.870		
Practices	P1	0.805	**0.898**	**0.564**
	P2	0.763		
	P3	0.799		
	P4	0.819		
	P5	0.793		
	P6	0.596		
	P8	0.597		

Model fit criteria: CR > 0.7, AVE > 0.5. All factor loadings were significant at *p* < 0.001, except for A3 (*p* > 0.05).

### 3.5 Structural model and hypothesis testing

To test the hypothesized relationships between the core constructs of knowledge, attitudes, and practices, a structural equation model (SEM) was employed. This analysis exclusively focused on the direct and indirect pathways connecting these three variables, as specified in hypotheses H1, H2, and H3.

#### 3.5.1 Model fit evaluation

**Figure 2:**
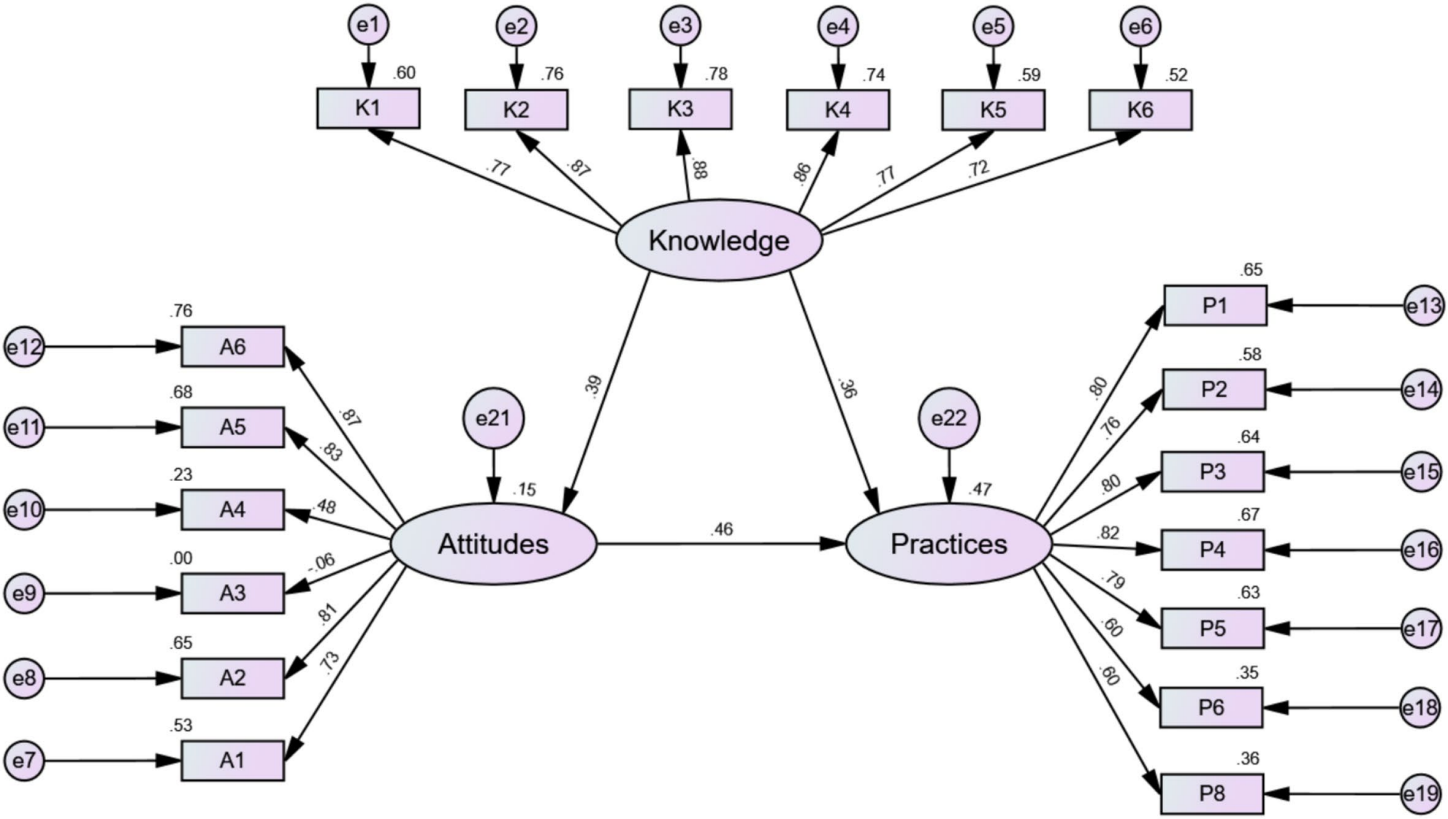
Fit results of the structural equation model for healthcare professionals’ CHS-DRG payment policy knowledge, attitudes, and practices.

The initial step was to evaluate the overall fit of the structural model. The model’s fit indices, presented in Table 6, indicated a strong fit with the data. The chi-square/degrees of freedom ratio (χ^2^/df = 3.029) was well within the acceptable range of < 5. Key incremental fit indices, including the Comparative Fit Index (CFI = 0.926) and the Tucker–Lewis Index (TLI = 0.915), exceeded the recommended 0.90 threshold. Furthermore, the Root Mean Square Error of Approximation (RMSEA = 0.075) was below the 0.08 cutoff. Collectively, these indices confirm that the proposed theoretical model structure adequately represents the relationships observed in the sample data. The final model is illustrated in Figure 2.

**Table 6:** Fit index for the structural equation model of CHS-DRG payment policy knowledge, attitudes, and practices for healthcare professionals.

Fit index	χ^2^/df	RMSEA	IFI	TLI	CFI
Fit standards	<5.00	<0.08	>0.90	>0.90	>0.90
Fit results	3.029	0.075	0.927	0.915	0.926

RMSEA, root mean square error of approximation; IFI, incremental fit index; TLI, Tucker–Lewis index; CFI, comparative fit index.

#### 3.5.2 Path analysis and hypothesis testing

The path analysis results, detailed in Table 7, revealed significant relationships among all three core constructs, providing support for all primary hypotheses.

**Table 7:** Path coefficients for the KAP model of CHS-DRG payment policy for healthcare professionals.

Paths			Estimate	S.E.	C.R.	*p*	Label
Attitudes	←	Knowledge	0.385	0.078	6.338	***	H1
Practices	←	Knowledge	0.360	0.140	6.693	***	H3
Practices	←	Attitudes	0.462	0.118	7.901	***	H2

*Hypothesis H1*: Knowledge exerted a significant and positive influence on Attitudes (*β* = 0.385, *p* < 0.001). This result supports H1, confirming that higher levels of policy knowledge are associated with more positive attitudes among healthcare professionals.

*Hypotheses H2 and H3*: Both Knowledge and Attitudes were found to be significant positive predictors of Practices. The path from Attitudes to Practices was strong and significant (*β* = 0.462, *p* < 0.001), supporting H2. Simultaneously, the direct path from Knowledge to Practices was also significant (*β* = 0.360, *p* < 0.001), supporting H3.

*Comparative Influence*: Notably, a comparison of the standardized path coefficients indicates that attitudes (*β* = 0.462) have a stronger direct effect on practices than knowledge (*β* = 0.360) does.

#### 3.5.3 Mediation analysis

Furthermore, a mediation analysis using bootstrapping was conducted to explore the indirect pathway through which knowledge influences practices via the mediating role of attitudes. The results, shown in Table 8, confirmed that attitudes function as a significant partial mediator. In addition to its significant direct effect, knowledge was found to have a significant indirect effect on practices (indirect effect β = 0.178, *p* < 0.001). This finding indicates that knowledge not only impacts practice directly but also shapes behavior indirectly by fostering more positive attitudes, which in turn leads to better implementation practices.

**Table 8:** Mediating effects of CHS-DRG payment policy knowledge and practices for healthcare professionals.

Parameter	Estimate	*p*
Indirect effects	0.178	<0.001
Direct effects	0.360	<0.001
Total effects	0.538	<0.001

## 4 Discussion

This study investigated the knowledge, attitudes, and practices (KAP) of healthcare professionals regarding the CHS-DRG payment policy within its primary implementation sites—the tertiary and secondary hospitals of Yunnan Province (16). The findings reveal that while professionals hold generally positive attitudes toward the reform, their knowledge and implementation practices remain at a moderate level (17). The analysis highlights two key insights: first, attitudes are a more powerful direct driver of practice than formal knowledge; and second, the influence of demographic characteristics is nuanced, with professional factors like experience and education level significantly impacting specific, distinct dimensions of KAP. These results offer crucial insights into the complex process of implementing major health policy reforms in real-world clinical settings (18).

The central finding of this research is that attitudes exert a stronger direct influence on practices (*β* = 0.462) than knowledge does (*β* = 0.360). This suggests that for a complex reform like CHS-DRG, simply disseminating information to increase knowledge is insufficient to ensure effective implementation (19). This phenomenon can be interpreted through the lens of organizational behavior theories: in the high-pressure, team-based environment of a hospital, an individual’s decision to adopt new procedures is heavily influenced by their personal assessment of the policy’s impact and the prevailing sentiment within their peer group (20). The data show that while professionals are receptive to the value-based goals of the policy, this optimism is tempered by significant concerns (21). This ambivalence is rooted in concrete fears: anxiety over financial implications for both the hospital and individual bonuses, and the perceived increase in workload stemming from demands for meticulous documentation and coding. This complex attitude—a blend of principled support and practical anxiety—appears to be the most critical determinant of their day-to-day actions (22).

Another noteworthy finding is the nuanced role of demographic characteristics. Rather than having a uniform effect, different factors distinctly influenced knowledge, attitudes, or practices. For instance, greater clinical experience, reflected in both age and years of work, was strongly associated with higher knowledge scores. This is an expected outcome, as senior professionals have had more exposure to policy training and administrative duties. However, this advanced knowledge did not uniformly translate into more positive attitudes or higher practice scores (23). This disconnect may be attributable to a “behavioral inertia” among more experienced professionals. While possessing greater knowledge, their long-standing clinical habits, honed under the fee-for-service model, may be more resistant to change. They might also perceive the standardized pathways inherent in DRGs as a threat to their clinical autonomy, fostering a less favorable disposition that decouples their knowledge from their practice (24).

Furthermore, education level presented a complex relationship with KAP, significantly influencing attitudes and practices but not knowledge. The finding that professionals with specialist (college) degrees held less positive attitudes than those with postgraduate degrees is particularly intriguing (25). This could suggest that postgraduate education, with its emphasis on research and critical appraisal, may foster a more skeptical stance toward top-down policy mandates, whereas vocational training may be more aligned with direct implementation and adherence (26).

These findings have direct and actionable implications that demand a multi-pronged and targeted approach from both national policymakers and local hospital administrators (27). For policymakers, enhancing the transparency of the DRG system’s financial mechanics is crucial to alleviate professional anxiety; this could involve publishing detailed simulation data on how clinical decisions impact reimbursement and establishing clear guidelines on the fair distribution of performance bonuses tied to DRG savings (28). For hospital administrators, the focus must shift from mere policy dissemination to a more sophisticated strategy of organizational engagement and tailored support:

### 4.1 Addressing attitudinal barriers

Recognizing that attitudes are the key driver of practice, leadership must establish regular, two-way communication forums where concerns about finances and workload can be openly discussed. Showcasing departmental successes and sharing best practices can transform abstract policy goals into tangible achievements, thereby fostering a more positive and collaborative climate (29).

### 4.2 Mitigating workload burdens

To address the concrete issue of increased workload, tangible resources are essential. This includes not only investing in user-friendly IT systems but also providing dedicated clinical coding support staff to assist physicians and optimizing the Electronic Health Record (EHR) with DRG-specific prompts and decision-support tools (30).

### 4.3 Implementing targeted training

A one-size-fits-all training approach is inefficient. Based on this study’s findings, training must be stratified. For experienced professionals with high knowledge but potential behavioral inertia, training should focus on case-based workshops and peer-led discussions to challenge long-standing habits (31). In contrast, junior professionals require training focused on foundational knowledge and practical coding skills (32). The expertise of highly knowledgeable groups like pharmacists should be leveraged by integrating them into multidisciplinary teaching teams, while professionals with postgraduate degrees, who may be more skeptical, could be engaged through participation in internal research and quality improvement projects evaluating the DRG implementation (33).

By adopting such targeted strategies, institutions can more effectively bridge the gap between policy knowledge and practical implementation.

## 5 Conclusion

This study’s primary contribution is the empirical demonstration that for complex, top-down health reforms like China’s CHS-DRG policy, managing the organizational and psychological climate is a critical lever for change. The research within Yunnan’s tertiary and secondary hospitals reveals that healthcare professionals’ attitudes, shaped by practical concerns over workload and finances, are the primary determinant of their implementation practices. While knowledge of the policy is a significant factor, its influence is secondary to the power of professional attitude, and the impact of demographic factors is specific and nuanced rather than uniform. Therefore, future implementation strategies should prioritize fostering a supportive environment that addresses these attitudinal barriers and tailor educational initiatives to the distinct needs of different professional subgroups. Further longitudinal and mixed-methods research is warranted to explore the causal mechanisms behind these drivers and to track the long-term impact of this pivotal reform.

## 6 Limitations of the study

Several limitations of this study must be acknowledged. First, the research scope was deliberately focused on tertiary and secondary hospitals in Yunnan Province; therefore, the findings may not be readily generalizable to primary care settings or to other Chinese provinces with different healthcare systems and policy implementation timelines. Second, the study’s cross-sectional design precludes the establishment of causal relationships between knowledge, attitudes, and practices. The reliance on self-reported data also introduces a potential for social desirability bias, wherein participants might overstate positive attitudes or compliance. Future research could overcome these limitations by employing a mixed-methods approach, incorporating qualitative interviews to triangulate findings, and using a longitudinal design to track changes over time. Finally, a methodological limitation should be noted: the ‘Attitudes’ construct demonstrated weak convergent validity (AVE < 0.5). While its internal consistency was acceptable, future studies should aim to develop and validate more robust scales to more accurately capture the nuances of this complex variable.

## Data Availability

The original contributions presented in the study are included in the article/supplementary material, further inquiries can be directed to the corresponding author.
