# Correction: Knowledge, attitudes, and practices regarding China's DRG payment policy among healthcare professionals in tertiary and secondary hospitals in Yunnan Province: a cross-sectional study

**DOI:** 10.3389/fpubh.2025.1741985

**Published:** 2025-12-11

**Authors:** Jian Yang, Dan Qin, Fan Li

**Affiliations:** 1 School of Pharmaceutical Sciences & Yunnan Provincial Key Laboratory of Pharmacology for Natural Products, Kunming Medical University, Kunming, Yunnan, China; 2 Yunnan Provincial Center for Drug Policy Research, Kunming, Yunnan, China; 3 College of Modern Biomedical Industry, Kunming Medical University, Kunming, Yunnan, China; 4 Technology Transfer Center, Kunming Medical University, Kunming, Yunnan, China

**Keywords:** CHS-DRG payment policy, value-based healthcare, knowledge, attitudes, and practices (KAP), structural equation modeling, hospital management, health policy

There was a mistake in Table 8 as published. The “Lower” and “Upper” confidence interval columns were incorrect and contradicted the “Estimate” column. For example, the “Indirect effects” Estimate 0.178 was not within its listed CI of (0.314, 0.649). The corrected Table 8 appears below.

**Table 8:** Mediating effects of CHS-DRG payment policy knowledge and practices for healthcare professionals.

**Parameter**	**Estimate**	***p***
Indirect effects	0.178	< 0.001
Direct effects	0.360	< 0.001
Total effects	0.538	< 0.001

There was a mistake in Table 1 as published. Minor rounding errors resulted in two categories summing to 100.1%. The corrected Table 1 appears below.

**Table 1:** Demographic characteristics of the participants.

**Variables**	**Variables**	***n***	**%**
Gender	Male	112	31.4
	Female	245	68.6
Age (years)	< 25	31	8.7
	26–35	176	49.3
	36–45	93	26.0
	>45	57	16.0
Professional title	Senior title/chief (e.g., Chief Physician)	16	4.5
	Associate Senior Title/Associate Chief (e.g., Associate Chief Physician)	46	12.9
	Intermediate Title (e.g., Attending Physician)	111	31.1
	Junior Title/Assistant (e.g., Resident Physician)	104	29.1
	Trainee/Intern	39	10.9
	Other	41	11.5
Position	Doctor	93	26.1
	Pharmacist	141	39.5
	Nurse	76	21.3
	Technician	20	5.6
	Medical insurance department staff	14	3.9
	Other	13	3.6
Education level	Master's degree and above	37	10.4
	Undergraduate	267	74.8
	Specialist	53	14.8
Hospital nature	Third level comprehensive hospitals (including traditional Chinese medicine hospitals)	212	59.4
	Secondary comprehensive hospitals (including traditional Chinese medicine hospitals)	124	34.7
	Traditional Chinese Medicine Specialized Hospital	12	3.3
	Community Health Service Center (including service stations)	2	0.6
	Township health centers (including clinics)	6	1.7
	Other	1	0.3
Years of work	0–1 years	9	2.5
	2–5 years	82	23.0
	6–10 years	110	30.8
	11–15 years	62	17.4
	16 years and above	94	26.3

① In the “Age (years)” category, the percentage for “36–45” was “26.1%”. This has been corrected to “26.0%”.

② In the “Hospital nature” category, the percentage for “Traditional Chinese Medicine Specialized Hospital” was “3.4%”. This has been corrected to “3.3%”.

In the published article, there was an error in the text. The text describing the findings for “Attitudes” by education level was incorrect and contradicted the data in Table 3.

A correction has been made to the section [*3.3, Analysis of KAP scores by demographic characteristics*, Paragraph 2]: Interestingly, professionals with specialist-level (college) degrees reported more positive attitudes than their counterparts with undergraduate or postgraduate degrees.

“Interestingly, professionals with specialist-level (college) degrees reported less positive attitudes (mean score: 12.41) than their counterparts with undergraduate (13.37) or postgraduate degrees (14.13).”

In the published article, there was a related error in the **Discussion** section, which also contradicted the data in Table 3.

A correction has been made to the section 4, **Discussion**, Paragraph 3: The finding that professionals with specialist (college) degrees held more positive attitudes than those with postgraduate degrees is particularly intriguing (25).

“The finding that professionals with specialist (college) degrees held less positive attitudes than those with postgraduate degrees is particularly intriguing (25).”

The original version of this article has been updated.

